# Qué camino tomar: la información errónea sobre la salud en las redes sociales^*^

**DOI:** 10.26633/RPSP.2021.58

**Published:** 2021-05-12

**Authors:** Wen-Ying Sylvia Chou, Anna Gaysynsky, Joseph N. Cappella

**Affiliations:** 1 Instituto Nacional del Cáncer Rockville Estados Unidos de América Instituto Nacional del Cáncer, Rockville, Estados Unidos de América.; 2 Instituto Nacional del Cáncer ICF Next Rockville Estados Unidos de América Instituto Nacional del Cáncer; ICF Next, Rockville, Estados Unidos de América.; 3 Escuela Annenberg de Comunicación de la Universidad de Pennsylvania Filadelfia Estados Unidos de América Escuela Annenberg de Comunicación de la Universidad de Pennsylvania, Filadelfia, Estados Unidos de América.

Se ha demostrado que las falsedades se propagan más rápido y llegan más lejos que la información correcta (1), y las investigaciones indican que la información errónea puede tener efectos negativos en el mundo real, como amplificar la controversia sobre las vacunas (2) y diseminar la recomendación de tratamientos oncológicos no comprobados (3). Por consiguiente, la información errónea sobre la salud en las redes sociales requiere con urgencia una mayor acción de quienes trabajan en la investigación y la práctica de la salud pública. Aquí definimos la “información errónea sobre la salud” como cualquier afirmación relacionada con la salud de un hecho que es falso sobre la base del consenso científico actual. Muchos otros tipos de información plantean un desafío para la comunicación de salud, incluidos los hallazgos contradictorios u opuestos, la evidencia cambiante y la información que suscita un alto grado de incertidumbre; sin embargo, estas cuestiones están fuera del ámbito de este artículo editorial, que se centra en información que es ostensiblemente falsa.

Responder a la información errónea es un desafío por muchas razones. Por ejemplo, los factores psicológicos, en particular las emociones y los sesgos cognitivos, pueden hacer que los esfuerzos directos por contrarrestar la información errónea proporcionando información correcta no sean eficaces. Esta puede ser la razón por la que las intervenciones, como recomendar artículos que presentan información correctiva, han demostrado tener una eficacia dispar (4). Otra cuestión es la dificultad para determinar quiénes están expuestos a la información errónea y llegar a ellos. La diversidad y el volumen de las redes sociales favorecen la creación y el mantenimiento de silos de información al facilitar a los usuarios la autocuración de los contenidos que reciben y encontrar contenidos similares mediante algoritmos automatizados. Estas características reducen la probabilidad de que las personas que forman parte de un grupo en el que circula información errónea estén expuestas a contenido que contradiga la opinión predominante en su red.

Como lo indica la evidencia acumulada, en ámbitos tan variados como las vacunas infantiles y la COVID-19, la información errónea generalizada sobre la salud puede tener consecuencias devastadoras, y las respuestas deben ser oportunas, estratégicas y basadas en la evidencia. Aquí proponemos cinco esferas de investigación que no se estudian lo suficiente y que deberían abordarse para mejorar las políticas y prácticas en respuesta a la información errónea sobre la salud (cuadro 1).

## Mejorar la vigilancia

Gran parte de la investigación realizada hasta la fecha se ha basado en el análisis transversal de contenidos de datos de las redes sociales (5) y, aunque esos tipos de estudios son importantes, la esfera de investigación debe avanzar hacia una comprensión más completa del entorno de información errónea de las redes sociales. Por ejemplo, muchos estudios se han centrado en Twitter^®^, pero otras plataformas populares, como WeChat^®^, Tumblr^®^, Reddit^®^ y Pinterest^®^, siguen sin estudiarse lo suficiente. Además, se necesita investigación para comprender mejor el contenido no textual, como imágenes, memes y videos que se encuentran en plataformas como Instagram^®^, TikTok^®^ y YouTube^®^, aprovechando el análisis visual asistido por computadora.

Las actividades de vigilancia también deben tener en cuenta los procesos complejos que afectan la difusión mediante el análisis sistemático de las dinámicas espaciales, temporales, de red y de plataformas múltiples de la propagación de la información errónea. Este conocimiento nos ayudaría a detectar las características esenciales de las plataformas, los contenidos y las redes que posibilitan o impiden la difusión de la información errónea.

**CUADRO 1.** Prioridades esenciales de investigación con respecto a la información errónea sobre la salud en las redes sociales

Mejorar la vigilancia	Comprender los factores psicológicos subyacentes	Evaluar las consecuencias en el mundo real	Centrarse en los grupos poblacionales vulnerables	Elaborar respuestas efectivas
Plataformas no estudiadas suficientemente Contenido visual Dinámica espacial, temporal, de redes y de múltiples plataformas de intercambio de información errónea	Emociones (p. ej., miedo, ansiedad) Identidad Sesgos cognitivos (p. ej., sesgo de confirmación)	Comportamientos y resultados de salud Actitudes (p. ej., apatía, confusión, desconfianza) Relaciones paciente-prestador Adopción de decisiones	Características de los grupos poblacionales vulnerables Grupos poblacionales objetivo de las intervenciones	Normas para determinar cuándo y cómo responder mejor Enfoques innovadores para penetrar en los silos Iniciativas de alfabetización mediática, de salud y de ciencias Medidas políticas

## Comprender los factores psicológicos subyacentes

También tenemos que aprovechar los marcos teóricos provenientes de las ciencias políticas, la psicología, la ciencia de las comunicaciones y otras ciencias sociales para examinar el papel que desempeñan las emociones, la cognición y la identidad respecto de la información errónea, y utilizar estos conocimientos para orientar las intervenciones. Por ejemplo, la tendencia humana hacia el sesgo de confirmación puede hacer que los esfuerzos encaminados a desmentir sean ineficaces, ya que la información correctiva puede ser vista como incompatible con la narrativa preferida y, por lo tanto, ser ignorada o negada. En situaciones en las que hay un fuerte sesgo de confirmación, las intervenciones basadas en la afirmación de valores podrían ser más eficaces.

De manera similar, aunque se han estudiado los efectos de las emociones en el procesamiento de la información errónea en el contexto de la política (6), se sabe menos acerca del papel que desempeñan las emociones cuando se trata de información errónea sobre la salud, a pesar de que los temas de salud pueden generar emociones fuertes, incluidos el miedo y la ansiedad. Para diseñar intervenciones fructíferas será esencial comprender más a fondo los factores psicosocioemocionales que impulsan la aceptación y el intercambio de información errónea, y la manera en que estos difieren en varios ámbitos (por ejemplo, la información sobre la política frente a la información sobre la salud).

## Evaluar las consecuencias de la información errónea

Se dispone de poca evidencia sobre la medida en que la exposición a la información errónea en línea afecta a los comportamientos, las actitudes, los conocimientos y los resultados relacionados con la salud a nivel individual o poblacional, o la forma en que la exposición a la información errónea se entrecruza con las disparidades de salud existentes.

Además, la información errónea puede tener consecuencias adicionales que, aunque difíciles de detectar, son igualmente insidiosas. Por ejemplo, la información errónea podría crear la impresión de que no hay consenso sobre un tema o que las fuentes oficiales de información no son creíbles, lo que podría generar sentimientos de apatía, confusión y desconfianza. Esto luego podría hacer que las personas pierdan interés en buscar información de salud, eludan la atención médica o tomen decisiones que son perjudiciales para su salud. Si bien es difícil vincular la actividad en línea con el comportamiento fuera de línea, se necesita más investigación empírica teóricamente informada para dilucidar el alcance pleno de las consecuencias que tiene en el mundo real la exposición a la información errónea.

## Centrarse en los más vulnerables

La investigación indica que la mayoría de las personas son susceptibles a la información errónea en algunos contextos y que los predictores sociodemográficos típicos de las disparidades de salud posiblemente no influyan en la vulnerabilidad a la información errónea. Por ejemplo, las personas con un alto nivel de escolaridad pueden ser igualmente vulnerables a la información errónea cuando se trata de temas que son fundamentales para su identidad (7). Detectar los factores que pueden aumentar la susceptibilidad a la información errónea (por ejemplo, una mentalidad conspirativa y la falta de acceso a la información de salud basada en la evidencia) permitiría dirigir mejor los recursos y adaptar mejor las estrategias.

Una vez que determinemos quién es el más vulnerable, se necesitarán métodos a fin de intervenir estratégicamente e influir en estos grupos. Por ejemplo, las intervenciones podrían utilizar fuentes de información que una comunidad vulnerable en particular considere creíbles para aumentar las probabilidades de aceptación de los mensajes. También se deberían realizar investigaciones para saber si las intervenciones deben dirigirse a las personas más influyentes de esos grupos vulnerables o centrarse en los que podrían estar menos integrados en el grupo y que aún podrían ser propensos a cambiar.

## Elaborar y probar respuestas efectivas

Un enfoque centrado simplemente en comunicar mensajes de salud basados en la evidencia o en desmentir ampliamente la información errónea posiblemente no sea suficiente. Se necesita investigación interdisciplinaria para elaborar estrategias adicionales y determinar el momento, la manera y el foro óptimos para responder a la información errónea. Si bien una respuesta reactiva podría ser eficaz en una situación, una respuesta proactiva (como la inoculación) puede ser vital en un contexto diferente. También es posible que la mejor respuesta sea no dar respuesta alguna, por ejemplo, si reconocer la falsedad en una corrección solo serviría para “darle oxígeno” y aumentar su propagación.

Además, se necesitan enfoques específicos para llegar con información correctiva a las personas que tienen información errónea. Los profesionales de la salud pública y los prestadores de atención de salud podrían intentar detectar y penetrar en los silos de información en línea donde la información errónea está extendida a fin de dar información basada en la evidencia, dirigir a los usuarios a fuentes creíbles o proporcionar contramensajes.

Por último, también es necesario realizar esfuerzos preventivos a nivel del sistema, como elaborar legislación que obligue a las plataformas de redes sociales a eliminar la información errónea, o dar incentivos para que estas adopten en mayor medida prácticas que dificulten más a los usuarios encontrar y transmitir información errónea. Otra estrategia de prevención importante consiste en aumentar la alfabetización mediática y en materia de salud y ciencias del público a fin de disminuir la vulnerabilidad a la información errónea. Este tipo de esfuerzos podría aumentar la conciencia sobre las técnicas (por ejemplo, seleccionar los datos según su conveniencia) que utilizan los agentes de la información errónea y aumentar la comprensión del público en cuanto a la incertidumbre inherente y la complejidad de la información en materia de salud y ciencias para suscitar un escepticismo saludable hacia las afirmaciones que son demasiado simplistas o sensacionalistas.

## Usar la investigación para orientar las políticas y las prácticas

Las prioridades de investigación que hemos esbozado deben orientar y mejorar las políticas y las prácticas encaminadas a hacer frente a la información errónea sobre la salud en las redes sociales, como las normas de moderación de contenidos utilizadas por las plataformas y las medidas para disipar los rumores adoptadas por los organismos de salud pública. A medida que se apliquen estas políticas e intervenciones, se necesitarán más investigaciones para evaluar sus efectos.

## Declaración.

Las opiniones expresadas por los autores son responsabilidad exclusivamente suya, y no debe interpretarse el contenido de este artículo como la postura oficial del Departamento de Salud y Servicios Humanos de Estados Unidos, los Institutos Nacionales de Salud o el Instituto Nacional del Cáncer; este tampoco refleja necesariamente los criterios ni la política de la *RPSP/PAJPH* y/o de la OPS.
